# Efficient Power Control Framework for Small-Cell Heterogeneous Networks

**DOI:** 10.3390/s20051467

**Published:** 2020-03-07

**Authors:** Qais Alsafasfeh, Omar A. Saraereh, Ashraf Ali, Luae Al-Tarawneh, Imran Khan, Adão Silva

**Affiliations:** 1 Department of Electrical Power and Mechatronics, Tafila Technical University, Tafila 11183, Jordan; qsafasfeh@ttu.edu.jo; 2 Department of Electrical Engineering, Hashemite University, Zarqa 13133, Jordan; eloas2@hu.edu.jo (O.A.S.); ashraf@hu.edu.jo (A.A.); 3 Communications Engineering Department, Princess Sumayya University for Technology, P.O.Box 1438, Amman 11941, Jordan; l.altarawneh@psut.edu.jo; 4 Department of Electrical Engineering, University of Engineering and Technology Peshawar, KPK P.O.Box 814, Pakistan; ikn.eup121@gmail.com; 5 Instituto de Telecomunicações (IT) and Departamento de Eletrónica, Telecomunicações e Informática (DETI), University of Aveiro, 3810-193 Aveiro, Portugal

**Keywords:** beyond 5G, heterogeneous networks, power control, small-cell

## Abstract

Heterogeneous networks are rapidly emerging as one of the key enablers of beyond fifth-generation (5G) wireless networks. It is gradually becoming clear to the network operators that existing cellular networks may not be able to support the traffic demands of the future. Thus, there is an upsurge in the interest of efficiently deploying small-cell networks for accommodating a growing number of user equipment (UEs). This work further extends the state-of-the-art by proposing an optimization framework for reducing the power consumption of small-cell base stations (BSs). Specifically, a novel algorithm has been proposed which dynamically switches off the redundant small-cell BSs based on the traffic demands of the network. Due to the dynamicity of the formulated problem, a new UE admission control policy has been presented when the problem becomes infeasible to solve. To validate the effectiveness of the proposed solution, the simulation results are compared with conventional techniques. It is shown that the proposed power control solution outperforms the conventional approaches both in terms of accommodating more UEs and reducing power consumption.

## 1. Introduction

The world with its ever-growing population and the continual increasing use of videos and the rise of Internet-of-things (IoT) is having an increase of wireless data traffic at a rate of over 50% per year per subscriber [1]. Addressing this demand, the wireless communication sector is evolving into the next generation of communication, which is the fifth-generation (5G) and wireless technology [2]. The previous generations of 2G, 3G, and the ongoing 4G networks are all based on microwave frequencies, unlike these generations, the 5G is based on millimeter waves which will provide a higher bandwidth, as a result, it will have the ability to support more users and provide higher data speeds of up to 10 Gbps and also lower the data traffic greatly.

Due to a rise in the traffic of wireless networks, it is becoming difficult for conventional cellular networks to accommodate and handle new users [3]. Moreover, the huge amount of mobile device traffic will also be tough for the cellular network to handle all on its own. One solution to this problem is to deploy a huge number of small cells in our environment [4]. But telecommunication companies are feeling reluctant on bearing the huge cost of buying, installing and maintenance of these small cells. By sending the user’s traffic over efficient small-cells BSs, telecommunication companies will need to install fewer small cells in our environment and it will also cause less traffic at the macro-cell.

However, deploying a large number of small-cell BS may not be the best solution and different users (vehicles, and pedestrians) may have different demands [5,6]. It is because increasing the number of small-cell BSs may result in a high amount of intercell interference. Moreover, the traffic demands within the cells may vary in practice due to the number of admitted users and their quality of service requirements. Thus, it is not feasible to simultaneously activate all the small-cell BSs at any particular time. The key theme of this paper is to use this intuitive fact to the advantage of network operators by reducing the amount of power consumed for operating small-cell BSs.

### 1.1. Related Work

Recently, there has been an upsurge in the interest in heterogeneous networks [7]. In this regard, the main techniques for exploiting heterogeneous networks have been deterministic or stochastic approaches [8]. Deterministic coverage allows us to calculate the necessary distance between co-channel cells and avoid interference [9]. Despite this approach’s effectiveness, this method sometimes becomes ineffective for highly dense urban scenarios where there are obstacles like building high traffic because of a huge number of users [10,11]. The deterministic method decreases rapidly due to such high fluctuations of cell load and the presence of skyscrapers that affects the network geometry negatively [12]. Therefore, stochastic geometry was introduced to predict the probabilistic parameters of randomly designed HetNet and the stochastic approach is showing better solutions [13].

In [14], the authors combined the software-defined networking approach with the small-cell deployments. They provided the concept of multiple TCP connections to ensure link reliability between the vehicles and small-cells. The authors of [15] provided a novel spatial phase coding technique for orthogonal frequency division multiplex (OFDM) systems. Their main object was to minimize inter-cell interference to enhance the reliability of mobile terminals. The results demonstrate that their proposed method reduces the bit-error-rate when compared to conventional techniques. Besides this, the incorporation of multi-antenna massive multiple-input-multiple-output (MIMO) networks is another important area of research for heterogeneous networks [16,17]. For instance, the authors of [18] consider full-duplex massive MIMO communication in heterogeneous networks and applied self-backhauling technique for small-cell networks.

Another work [19] consider a 3D MIMO heterogeneous network and perform the trial results. Their measurement shows the promising features of the heterogeneous network when combined with 3D MIMO. From the perspective of performance evaluation, the authors of [20] investigated the outage performance of heterogeneous networks. Waidhuba et al. in [21] aims to improve the performance of wireless backhaul links in massive MIMO heterogeneous networks. They also study the spectral efficiency of the system and identify the different requirements and connectivity issues of backhaul links. A novel interference alignment technique was proposed by the authors of [22]. They investigate small-cell based heterogeneous networks and identified the problem of inter-cell interference. Their proposed interference alignment technique improves upon the existing baseline approaches. A two-stage device-to-device (D2D) communication technique was proposed by the authors of [23]. They highlighted that the problem of computation resource management could become critical for large-scale heterogeneous networks. Thus, they provided D2D-assisted approach to balance the computation resource in the small-cells which significantly improves the performance of the network. They used a hybrid coordinated beamforming technique for signal transmission. They identified the key outage constraints on the performance of such networks. An energy-efficient and spectral efficient tradeoff for the small-cell backhaul networks was provided by the authors of [24,25]. The authors claimed that there is a need to find the optimal balancing point between the tradeoff of spectral and energy efficiency. Energy-efficient scheduling techniques were proposed by the authors of [26,27] for conventional small-cell cellular networks and unmanned aerial vehicle (UAV) assisted small-cell communications [28]. In a similar manner, the user association problem was investigated by the authors of [29]. It was shown that significant performance gains could be obtained by jointly optimizing the resources and associating the users.

### 1.2. Motivation and Contribution

Though there has been considerable with regards to the development of efficient techniques for heterogeneous networks, the power controlling aspect of such networks has received little attention. This is particularly because small-cell base station (BS) is considered to be operating at all the time in the heterogeneous network, which may not be always true. We propose that small-cell BSs can be controlled efficiently to reduce the power consumption of such networks. Thus, to this end, our work provides the following key contribution to the state-of-the-art:

1. We develop a heterogeneous network having multicast groups of user equipments (UEs) served by different small-cell BSs. The small-cell BSs can be controlled by macro-cell BS to switch on and off based on the network demands.
2. Due to the sparse nature of the formulated problem, a small-cell BS switching off mechanism has been introduced to control the power of the network. When the problem becomes infeasible, the admission control policy has been introduced to meet the quality of service requirements of the UEs in the network.
3. Detailed comparison with existing state-of-the-art approaches (coordinated beamforming and membership deflation) has been provided. The results show that the proposed power control solution outperforms the conventional techniques significantly in terms of reduction in power consumption and the capacity of admitted UEs.

### 1.3. Organization

The remainder of the paper is organized as follows. Section 2 provides the details of the system model. Section 3 presents the problem transformation steps and proposed a power control algorithm. In Section 4, the simulation results and relevant discussion has been provided. Finally, Section 5 provides some concluding remarks along with the main future research directions.

## 2. System Model

**Figure 1:**
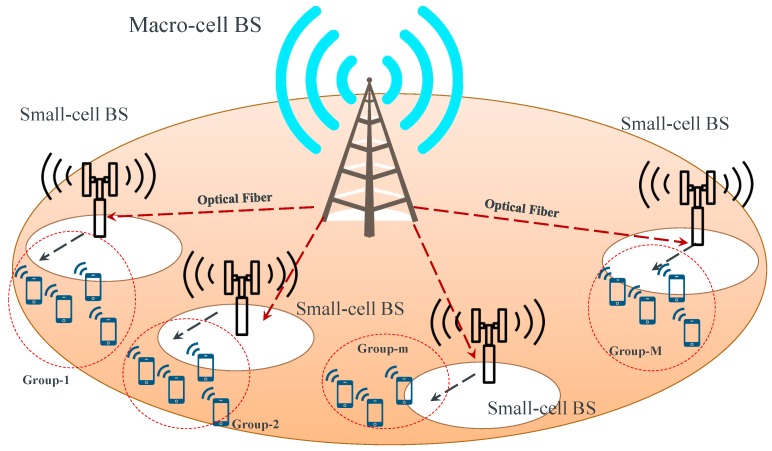
System Model.

We consider a heterogeneous network having a single macro-cell and multiple small cells, as shown in Figure 1. In total, we assume that there are *L* small cells, whereby, each small-cell BS is equipped with $N_{l}$ antennas. We also consider that each small-cell is serving *K* UEs that are equipped with single antennas. The UEs are divided into *M* nonoverlapping multicast groups based on their quality of service requirements. For instance, the UEs could be categorized into groups based on their application and data rate needs. However, since the main purpose of this work is to provide a solution for power control problem in small-cell network, the issue of multicast group formation is beyond the scope of this work. The channel from the *l*-th small-cell to the *k*-th UE is represented as $h_{kl}\in C^{N_{t}}$. The transmitted signal from the small-cell is written as

(1) $$\begin{array}{c} x_{l}=\sum_{m=1}^{M}v_{lm}s_{m}, \end{array}$$

where $v_{lm}\in C^{N_{t}}$ is the transmit beamforming vector to the UEs from the *l*-th small-cell. Moreover, $s_{m}\in C$ is the symbol, where it is considered that $E[|s_{m}{|}^{2}]=1$. The transmit power of the *l*-th small-cell is constrained by the maximum power, i.e., $P_{l}$. This constrained on the beamforming vector is represented as

(2) $$\begin{array}{c} V=\{v_{lm}\in C^{N_{l}}:\sum_{m=1}^{M}|v_{lm}{|}_{2}^{2}\leq P_{l}\} \end{array}$$

As a result of the transmission, the received signal at the *k*-th UE in *m*-th group can be written as

(3) $$\begin{array}{c} y_{km}=\sum_{l=1}^{L}h_{kl}^{H}v_{lm}s_{m}+\sum_{i\neq m}\sum_{l=1}^{L}h_{kl}^{H}v_{li}s_{i}+n_{k} \end{array}$$

where all the users are considered to apply single user detection, thus, $s_{m}$ and $n_{k}$ are mutually independent. Moreover, $n_{k}$ is the additive white Gaussian noise with $\lambda_{k}^{2}$ as variance and 0 mean. Based on this, the signal-to-intereference-and-noise ratio (SINR) is written as

(4) $$\begin{array}{c} \Omega_{k,m}\left(v\right)=\frac{v_{m}^{H}\delta_{k}v_{m}}{\sum_{i\neq m}v_{i}^{H}\delta_{k}v_{i}+\lambda_{k}^{2}} \end{array}$$

where $N=\sum_{l=1}^{L}N_{l}$ is the vector dimension for $\delta_{k}=h_{k}h_{k}^{H}$. Also, the channel vectors and beamforming vectors having beamforming coefficients are, respectively, given as

(5) $$\begin{array}{c} h_{k}={\left[h_{k1}^{H},h_{k2}^{H},\ldots ,h_{kL}^{H}\right]}^{H}, \end{array}$$

and

(6) $$\begin{array}{c} v_{m}=\left[v_{m1}^{H},v_{m2}^{H},\ldots ,v_{mL}^{H}\right]. \end{array}$$

We now focus our attention on the power-control problem in heterogeneous networks. One way for controlling the power of the network for macro-cell BS is to reduce the activity of small-cell BSs. Specifically, based on the variations in data traffic and quality of service demands, the macro-cell BS can switch off the small-cell BSs. This can put the inactive small-cell BSs in dormant mode, thereby, reducing the consumed power at the BSs. Let us represent the power consumption from small-cell BSs to UEs as $p_{1}$ and from macro-cell BS to small-cell BS as $p_{2}$. Then, the total consumed power can be denoted as

(7) $$\begin{array}{c} p\left(v\right)=p_{1}\left(v\right)+p_{2}\left(v\right) \end{array}$$

For any value of drain efficiency coefficient, $\mu_{l}>0$, the  consumed power $p_{1}\left(v\right)$ is given as

(8) $$\begin{array}{c} p_{1}\left(v\right)=\sum_{l=1}^{L}\sum_{m=1}^{M}\frac{1}{\eta_{l}}{\left|v_{lm}\right|}_{2}^{2}. \end{array}$$

Similarly, $p_{2}\left(v\right)$ is defined as

(9) $$\begin{array}{c} p_{2}\left(v\right)=\sum_{l=1}^{L}P_{l}^{c}I\left(S\left(v\right)\cap V_{l}\neq \emptyset \right). \end{array}$$

It is worth mentioning that $I(S\left(v\right)\cap V_{l}\neq \emptyset )$ represents whether the *l*-th small-cell BS is switched off or on and S(v) is the support vector of *v*. This is called an indicator function and takes a value of 0 when a particular small-cell BS is off. Additionally, $P_{l}^{c}$ is the passive optical power consumption for the *l*-th small-cell BS. Moreover, $V_{l}$ is defined as

(10) $$\begin{array}{c} V_{l}\in \left\{M\sum_{l=1}^{L-1}N_{l}+1,\ldots ,M\sum_{l=1}^{L}N_{l}\right\} \end{array}$$

For a pre-specified quality of service requirements of different UEs in the network, i.e., $\left(\omega_{1},\omega_{2},\ldots \omega_{K}\right)$, it is important to control the power subject to these constraints. Thus, we have the following constraint on the quality of service of each UE

(11) $$\begin{array}{c} \Omega_{k,m}\left(v\right)\geq \omega_{k}. \end{array}$$

After replacing the corresponding expressions, the above constraint can be rewritten as the quadratic constraint which is given by

(12) $$\begin{array}{c} Z_{k,m}\left(v\right)=\omega_{k}\left(\sum_{i\neq m}v_{i}^{H}\delta_{k}v_{i}+\lambda_{k}^{2}\right)-v_{m}^{H}\delta_{k}v_{m}\leq 0. \end{array}$$

Thus, the power control problem can be written as

(13) $$\begin{array}{c} \min(p_{1}\left(v\right)+p_{2}\left(v\right)) \end{array}$$

$$\begin{array}{c} s.t.\;\;Z_{k,m}\left(v\right)\leq 0 \end{array}$$

## 3. Proposed Power Control Solution

In this section, we provide a solution to the formulated problem using efficient UE admission control.

### 3.1. Problem Transformation

From (12), one can observe that it is nonconvex quadratic function. Due to this reason, the problem in (13) can be categorized as a nonconvex combinatorial problem. However, it is worth noting that due to the on-off switching of small-cell BSs, the solution for the formulated problem has group sparsity [30]. This means that if any *l*-th small-cell BS is switched off, the corresponding beamforming coefficients $v_{l}$ would also be set to 0. Therefore, we introduce the weighted mixed norm to introduce group sparsity and relax the combinatorial composite function. Thus, for any weight of beamforming coefficient group $\rho_{l}>0$, we have

(14) $$\begin{array}{c} J\left(v\right)=\sum_{l=1}^{L}\rho_{l}{\left|v^{{}^{\prime }}\right|}_{2}. \end{array}$$

Besides, due to the constraint of quality of service, it is possible that the formulated problem becomes infeasible for a large number of UEs. In such a situation, the design problem would be to maximize the number of users that can be supported via admission control. Thus, we can add an auxiliary variable $x_{k}$ such that maximizing the number of users in the network is equivalent to minimizing the number of nonzero $x_{k}$. Thus, the problem can be reformulated as

(15) $$\begin{array}{c} \min_{v,x}{\left|x\right|}_{0} \end{array}$$

$$\begin{array}{c} s.t.\;\;Z_{k,m}\left(v\right)\leq x_{k} \end{array}$$

Note that when $x_{k}=0$, it means that *k*-th UE can be addition and vice versa. Thus, the solution of (15) has individual sparsity and sparsity increases for a larger number of admitted UEs.

To solve the previously mentioned problems, we use $l_{p}$ minimization to find the approximation of both the objective functions. We adopt ${\left||x|\right|}_{p}^{p}$ and induce a quadratic form to have a more sparser solution. Specifically, we adopt following smoothed version of ${\left||x|\right|}_{p}^{p}$:

(16) $$\begin{array}{c} f_{p}\left(x;\varepsilon \right)\in \sum_{i=1}^{m}{\left(x_{i}^{2}+\varepsilon^{2}\right)}^{\frac{p}{2}}. \end{array}$$

Thus, the power control problem can now be written as

(17) $$\begin{array}{c} \min_{v}\sum_{l=1}^{L}\rho_{l}\left(\right|v^{{}^{\prime }}{{|}_{2}^{2}+\varepsilon^{2})}^{\frac{p}{2}} \end{array}$$

$$\begin{array}{c} s.t.Z_{k,m}\left(v\right)\leq 0 \end{array}$$

It can be seen that the induced sparse beamformers would help select the appropriate small-cell BS. Moreover, the resulting problem is a quadratic optimization problem which can be solved in a feasible manner.

For admission control, we take advantage of smoothed $l_{p}$-norm approach to approximate the objective function. Thus, we have

(18) $$\begin{array}{c} \min_{v,x}\sum_{k=1}^{K}{\left(x_{k}^{2}+\varepsilon^{2}\right)}^{\frac{p}{2}} \end{array}$$

(19) $$\begin{array}{c} s.t.\;\;Z_{k,m}\left(v\right)\leq x_{k} \end{array}$$

Note that the above formulation would help induce an individual sparsity, thereby, helping in UE admission control.

### 3.2. Proposed Solution

In this section, we apply semidefinte relaxation technique to solve the nonconvex problems in (17) and (18). We first modify the quality of service constraint in (12) by letting $Q_{m}=v_{m}^{H}v_{m}$ as

(20) $$\begin{array}{c} W_{k,m}\left(Q\right)\leq 0 \end{array}$$

where

(21) $$\begin{array}{c} W_{k,m}\left(Q\right)=\omega_{k}\left(\sum_{i\neq m}Tr(\delta_{k}Q_{i})+\lambda_{k}^{2}\right)-Tr\left(\delta_{k}Q_{m}\right) \end{array}$$

Moreover, for the selection of appropriate small-cell BS, the power constraint in (2) is given as

(22) $$\begin{array}{c} Q=\{Q_{m}\in C^{N}:\sum_{m=1}^{M}Tr(C_{lm}Q_{m})\leq P_{l}\} \end{array}$$

Now, by dropping the rank-one constraint, as per the principle of semidefinite relaxation, we rewrite problem (17) as

(23) $$\begin{array}{c} \min_{Q}\sum_{l=1}^{L}\rho_{l}{\left(\sum_{m=1}^{M}Tr\left(C_{lm}Q_{m}\right)+\varepsilon^{2}\right)}^{\frac{p}{2}} \end{array}$$

$$\begin{array}{cc} s.t. & \;\;W_{k,m}\left(Q\right)\leq 0\\ \; & Q_{m}\geq 0 \end{array}$$

In a similar manner, the rank-one constraints in (18) can be dropped. Thus, the new problem becomes

(24) $$\begin{array}{c} \min_{Q,x}\sum_{k=1}^{K}{\left(x_{k}^{2}+\varepsilon^{2}\right)}^{\frac{p}{2}} \end{array}$$

$$\begin{array}{cc} s.t. & \;\;W_{k,m}\left(Q\right)\leq x_{k}\\ \; & Q_{m}\geq 0 \end{array}$$

Using the induced sparse approximation, we can extract sparsity structure as per following expression:

(25) $$\begin{array}{c} |v_{l}^{{}^{\prime }}{|}_{2}=\sqrt{\sum_{m=1}^{M}Tr(C_{lm}Q_{m})} \end{array}$$

Letting the sum of channel gains as $\kappa_{l}=\sum_{k=1}^{K}\left|\right|h_{kl}{\left|\right|}_{2}^{2}$ for any *l*-th small-cell BS, we adopt the following small-cell BS switching off selection criteria to control the network power

(26) $$\begin{array}{c} \Delta_{l}=\sqrt[{}]{\frac{\mu_{l}\kappa_{l}}{P_{l}^{c}}}{\left(\sum_{m=1}^{M}Tr(C_{lm}Q_{m}^{})\right)}^{0.5} \end{array}$$

From the above expression, one can observe that any small-cell BS with lower drain efficiency $\mu_{l}$, higher optical power consumption $P_{l}^{c}$, lower channel gain $\kappa_{l}$, and lower beamforming gain $v_{l}^{{}^{\prime }}$ would have higher priority for switching off. However, we introduce a limit of $J_{0}$ to the total number of small-cell BSs that can be switched off at any moment. In order to find $J_{0}$, we need to solve the following problem

(27) $$\begin{array}{c} F\left(A^{\left[i\right]}\right):findQ_{1}^{\left[i\right]},\ldots ,Q_{M}^{\left[i\right]} \end{array}$$

$$\begin{array}{cc} s.t. & \;\;W_{k,m}\left({\left\{Q_{m}^{\left[i\right]}\right\}}_{m}\right)\leq 0\\ \; & Q_{m}^{\left[i\right]}\geq 0 \end{array}$$

where

(28) $$\begin{array}{c} Q_{m}^{\left[i\right]}\in C^{\left(\sum_{l\in A^{\left[i\right]}}N_{l}\right)\times \left(\sum_{l\in A^{\left[i\right]}}N_{l}\right)} \end{array}$$

Is the transmit power constraint for the set of switched on small-cell BS which is given as

(29) $$\begin{array}{c} A^{\left[i\right]}=\left\{\zeta_{i+1},\ldots ,\zeta_{L}\right\}. \end{array}$$

We now solve the following size reduced semidefinite programming problem

(30) $$\begin{array}{c} \min_{Q^{\left[J_{0}\right]}}\sum_{l\in A}\sum_{m=1}^{M}\frac{1}{\mu_{l}}Tr\left(C_{lm}Q_{m}^{\left[J_{0}\right]}\right) \end{array}$$

$$\begin{array}{cc} s.t. & \;\;W_{k,m}\left(Q^{\left[J_{0}\right]}\right)\leq 0\\ \; & Q_{m}^{\left[J_{0}\right]}\geq 0 \end{array}$$

The above problem can be solved using the interior point method.

If the problem is not feasible as per the criterion of [31] (Section IV), it would be necessary to perform UE admission control. By this way, the total number of supported UEs could be improved in the network. Let that $N_{0}$ represents the total number of UEs that be removed to support the quality of service requirements of other UEs. To find the value of $N_{0}$, it would be important to solve the following problem

(31) $$\begin{array}{c} F\left(S^{\left[i\right]}\right):find{\left\{Q_{m}\right\}}_{m} \end{array}$$

$$\begin{array}{cc} s.t. & \;\;W_{k,m}\left({\left\{Q_{m}\right\}}_{m}\right)\leq 0\\ \; & Q_{m}\geq 0 \end{array}$$

where the set of admitted UEs and the set of multicast groups is, respectively, given as

(32) $$\begin{array}{c} S^{\left[i\right]}=\left\{\zeta_{i+1},\ldots ,\zeta_{K}\right\} \end{array}$$

and

(33) $$\begin{array}{c} M^{\left[i\right]}=\left\{m:G_{m}\cap S^{\left[i\right]}\neq \emptyset \right\} \end{array}$$

Once the set of admitted UEs is known, the power control problem can be solved in a straightforward manner as given in Algorithm 1.

**Algorithm 1:** Power Control of Network. **Require:** Initial the system parameters.  **if** Sparse solution is feasible **then**   Obtain $Q_{m}^{*}$, sort $\Delta_{l}$ in ascending order.
   Initialize $J_{up}=L$, $J_{low}=0$, and iteration index i=0.   Set $i=\frac{J_{up}+J_{low}}{2}$.   Solve $F(A^{\left[i\right]})$.   Set $J_{low}=i$ if feasible, otherwise, set $J_{up}=i$.   Until $J_{low}-J_{up}=-1$, obtain $J_{0}=J_{low}$ and get beamforming vectors as $A^{*}=\left\{\zeta_{J_{0}+1},\ldots \zeta_{L}\right\}$.  **else if** Infeasible **then**   Obtain $x^{*}$, sort $x_{l}$ in descending order.   Initialize $N_{up}=K$, $N_{low}=0$, and iteration index i=0.   Set $i=\frac{N_{up}+N_{low}}{2}$.   Solve $F(S^{\left[i\right]})$.   Set $N_{low}=i$ if feasible, otherwise, set $N_{up}=i$.   Until $N_{low}-N_{up}=-1$, obtain $N_{0}=N_{up}$ and get admitted UEs as $S^{*}=\left\{\zeta_{N_{0}+1},\ldots \zeta_{K}\right\}$.  **end if**


## 4. Numerical Results

This section provides numerical results and presents a relevant discussion. We compare the results with two renowned schemes: coordinated beamforming and membership deflation approach. Besides this, the results are compared with the exhaustive search process which is more complex and not scalable for large-scale networks. Unless mentioned otherwise, the values of different simulation parameters are given in Table 1.

**Figure 2:**
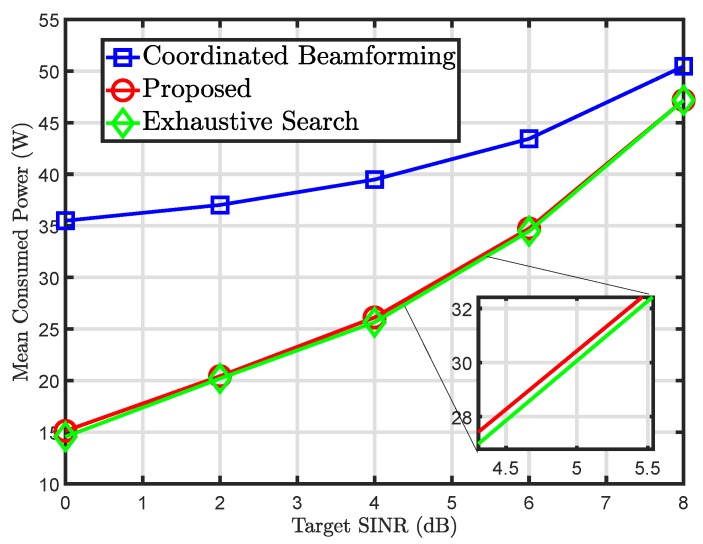
Mean power consumption versus target signal-to-intereference-and-noise ratio (SINR).

Figure 2 shows the mean power consumption for increasing values of target SINR. It can be noted that an increase in the target SINR results in more power consumption of the small-cell BSs. However, when compared to the conventional coordinated beamforming approach, the proposed solution outperforms significantly. Besides, the proposed algorithm also closely follows the exhaustive search process which indicates the feasibility of the proposed solution. Since the exhaustive search approach does not scale well with an increase in the network size, the proposed solution is a better option among the two.

**Figure 3:**
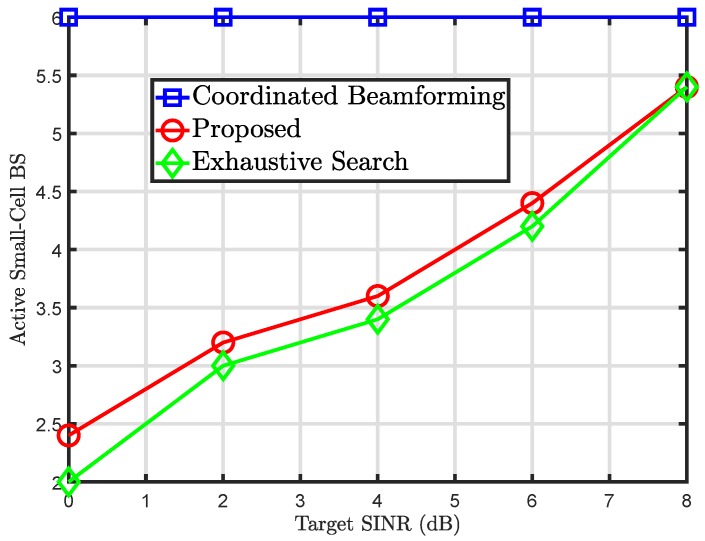
Number of active small-cell base stations (BSs) against target SINR.

Figure 3 shows a number of active small-cell BSs against the increasing values of target SINR. It can be seen from the figure that to match the target SINR values, it becomes important to activate a larger number of small-cell BS. Due to this reason, the active number of small-cell BS increases linearly with an increase in target SINR. However, one can note that the coordinated beamforming approach performs worst among both the proposed solution and the exhaustive search approach. The proposed solution again closely follows the exhaustive search approach for smaller values of SINR but overlaps for larger values for target SINR. This again shows the feasibility of the proposed solution since a smaller number of BSs needs to be activated to meet the quality of service requirements of the UEs.

**Figure 4:**
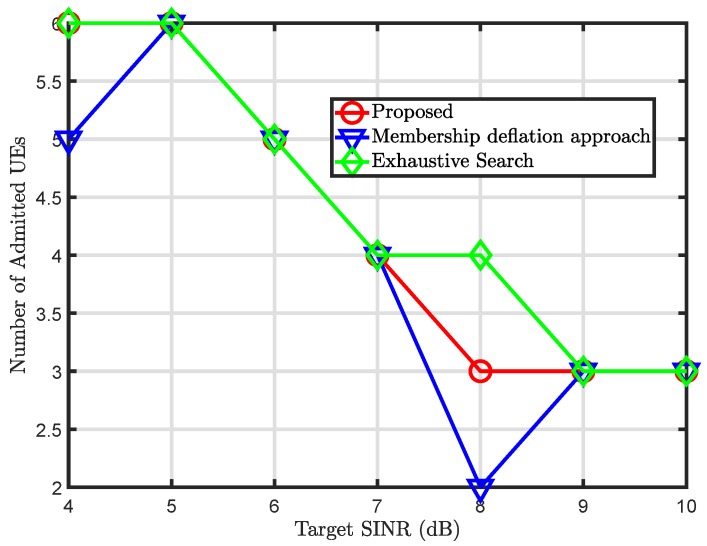
Total admitted user equipments (UEs) versus target SINR.

Figure 4 illustrates the comparison of the proposed solution against the membership deflation approach in terms of a number of admitted UEs in the network. It can be seen from the figure that to meet the target SINR demands of the UEs, it is important to keep the admitted UEs at a minimum. This is the reason why the total number of admitted UEs decrease with an increase in the values of target SINR. However, our proposed solution tries to maximize the number of admitted UE while meeting the quality of service demands of the UEs. When compared to the proposed solution, the membership deflation approach does not perform well. The membership deflation approach allows a limited number of UEs to be admitted to the network against the same value of SINR. By contrast, the proposed solution outperforms the membership deflation approach and closely follows the exhaustive search technique. This clearly indicates the proposed solution is not only good at controlling the power of the network but also improves the overall capacity of the network to accommodate a larger number of UEs.

## 5. Conclusions and Future Work

Heterogeneous networks are going to play a key role beyond 5G networks. In this regard, a feasible solution to the power control problem of small-cell heterogeneous networks is provided in this work. More specifically, we have developed an optimization framework that allows the small-cell BSs to switched off when the traffic demands are not critical. The sparse structure of the problem helps in guiding the selection of small-cell BSs that needs to be switched off and improve user admission. The simulation results demonstrate the effectiveness of the proposed solution. When compared to the conventional coordinated beamforming approach, it has been shown that the proposed approach requires a lesser number of active small-cell BSs. Furthermore, the mean power consumption of a coordinated beamforming approach is also 20 W higher than the proposed approach. Besides, it has also been shown that the proposed solution improves the number of admitted UEs when compared to the membership deflation approach. Thus, it has been shown that the proposed solution outperforms the said approaches in terms of reduction in power consumption and improvement in user admission.

Though the proposed solution for power controlling significantly improves the performance of the network, many extensions can further improve the state-of-the-art. For instance, the proposed solution can be extended for imperfect and outdated channel estimation. This can have an impact on the sparsity of the problem which requires further investigation. Moreover, the proposed solution can be extended for the delay-sensitive mobile network. In this case, the user admission control would require a much more robust framework. These problems are interesting and challenging and left for future work.

## Figures and Tables

**Table 1:** Simulation parameters and their values.

Simulation Parameters	Value
$P_{l}^{c}$	5.6 W
$\lambda_{k}$	1
$\eta_{l}$	0.25
ϵ	0.001
Number of small-cell BS	6
Total multicast groups	2

## Abbreviations

The following abbreviations are used in this manuscript:

5G	Fifth-generation
SINR	Signal-to-intereference-and-noise ratio
IoT	Internet-of-things
UAV	Unmannaed aerial vehicle
BS	Base station
MIMO	Multiple-input-multipl-output
UE	User equipment
